# Classification of Oral Squamous Cell Carcinoma from Histopathological Images Using a Hybrid Deep Learning Model

**DOI:** 10.3390/diagnostics16182885

**Published:** 2026-09-08

**Authors:** Furkan Talo, Ahmet Bedri Ozer

**Affiliations:** Department of Computer Engineering, Firat University, 23119 Elazig, Turkey; bedriozer@firat.edu.tr

**Keywords:** oral cancer, histopathology, deep learning, ViT, CNN, classifiers

## Abstract

**Background/Objectives:** Oral squamous cell carcinoma (OSCC) has high mortality rates and leads to serious health problems when diagnosed late. This situation is considered a public health problem. Histopathological examination, which is an important point in the diagnosis of the disease, is a time-consuming and manual process that requires expertise. **Methods:** This study presents a deep learning-based approach for the automated classification of normal oral epithelium and oral squamous cell carcinoma (OSCC) from histopathological images. The performance of state-of-the-art architectures such as Vision Transformer (ViT), CLIP, ConvNextV2, Deit, and Dinov2 was comparatively analyzed. Based on the results, a hybrid architecture combining the strengths of the models is proposed. **Results:** In experiments conducted on a dataset of 696 histopathological images, the ConvNextV2 and ViTL16 architectures stood out among the basic models with accuracy rates around 84%. However, the most significant contribution of this study is the proposed method, which combines the global context capability of Transformer-based models with the local feature extraction power of CNN-based models using the Efficient Channel Attention (ECA)—Gated Features model. This hybrid model, created by integrating the ViTL16, Dinov2, and ConvNextV2 architectures, achieved 89.95% accuracy, 89.82% F1 score, and 89.95% sensitivity with a KNN classifier, outperforming the baseline models in the literature. **Conclusions:** The results obtained demonstrate that the fusion of multiple architectures increases diagnostic reliability in medical image analysis and can assist pathologists as a decision support mechanism.

## 1. Introduction

Oral cancer is a malignant tumor that develops as a result of uncontrolled cell proliferation and causes damage by spreading to surrounding tissues. This uncontrolled division results in abnormal tissue growths in the oral cavity that clinically resemble ulcerative lesions. This disease, the sixth-most common cancer globally, is said to be more prevalent in men over 50 years of age compared to women [1,2]. The average age of diagnosis is estimated to be 63, although it is also reported in the literature that it can occur in younger individuals [2]. When examined on a global scale, oral cancer is considered a significant public health problem for countries undergoing economic transition [3]. For example, in India, an average of 77,000 new cases and 52,000 deaths are reported annually, accounting for approximately one-third of global cases [4], representing 30% of all cancer types [5]. This increase in case numbers poses a significant challenge to public health and negatively impacts the quality of healthcare services [6].

When examining the causes of the disease, poor oral hygiene [7] and excessive use of tobacco and alcohol [8,9] are among the main causes. Chewing a mixture of betel leaf, areca nut, and tobacco, which is common in regions such as India and has addictive effects, is also a significant risk factor [10,11]. Sharp tooth structures causing chronic trauma, immune system deficiencies, and alterations in genetic material are other factors playing a role in pathogenesis [12]. Histopathologically, oral cancer is classified into three main categories: oral squamous cell carcinoma (OSCC), verrucous carcinoma, and minor salivary gland carcinomas. Ninety percent of cases are caused by OSCC, which develops as a result of squamous cells undergoing mutation and becoming atypical [13]. Verrucous carcinoma accounts for 3 to 5% of all cases, while minor salivary gland carcinomas are rarer [14].

The progression of the disease occurs in different stages, and physicians generally conduct the diagnostic process in two phases: clinical and pathological [15]. To increase survival rates, the spread of malignant tumor cells can be stopped with early diagnosis. Therefore, early diagnosis is critical in improving survival rates.

Histopathological examination plays a central role in the diagnosis of oral cancer. This study focuses on the automated classification of histopathological images using artificial intelligence-based computational approaches. Image processing techniques are widely used in the analysis of images obtained from different scanning models. The images obtained are pre-processed using histogram equalization techniques and various filtering algorithms for noise reduction and image smoothing. Although not common, invasive detection methods using small devices are also available in the literature [16].

Artificial intelligence (AI) techniques, which optimize performance through learning and prediction capabilities, are effectively used in the detection of oral cancer, as in many disciplines, and offer high success rates. AI approaches, with their wide range of algorithms, provide a powerful platform that enables accurate and reliable results [17]. In this study, an attempt was made to detect oral diseases using the developed hybrid model. The innovative aspects of the proposed model and the contributions of the study to the literature can be listed as follows.

- This study presents a deep learning-based approach for the automated classification of normal oral epithelium and OSCC using histopathological images. In this study, a learnable Efficient Channel Attention (ECA)-gate-based model was developed to combine features obtained from pre-trained deep learning architectures. The proposed structure combines the features obtained from different models and automatically suppresses less useful features in terms of classification, thereby enabling a discriminative representation.
- In the proposed method, the ECA mechanism is applied at the level of vectorized deep features rather than on CNN intermediate layers, which are commonly used in the literature. This design choice results in a structure with fewer parameters, lighter computation, and greater stability during training compared to SE-based MLP approaches.
- The ECA-gate mechanism is trained in a controlled manner through an auxiliary linear classification head, rather than with an intuitive or fixed weighting. This approach enables learning that is directly sensitive to class separation, transforming the method into a learnable feature selection and rearrangement paradigm beyond classical feature fusion approaches.
- The effectiveness of standard deep learning architectures was classified using different classical machine learning classifiers. This comprehensive analysis demonstrates that the proposed representation is not specific to a particular classifier and has general validity.
- The study achieved a high accuracy value of 89.95%. This value is higher than the seven different CNN and ViT architectures used for comparison in the study.

The Section 2 is presented in the continuation of the article. This is followed by Section 3, and then Section 4. Finally, the study concludes with the Section 5.

## 2. Materials and Methods

### 2.1. Dataset

**Figure 1:**
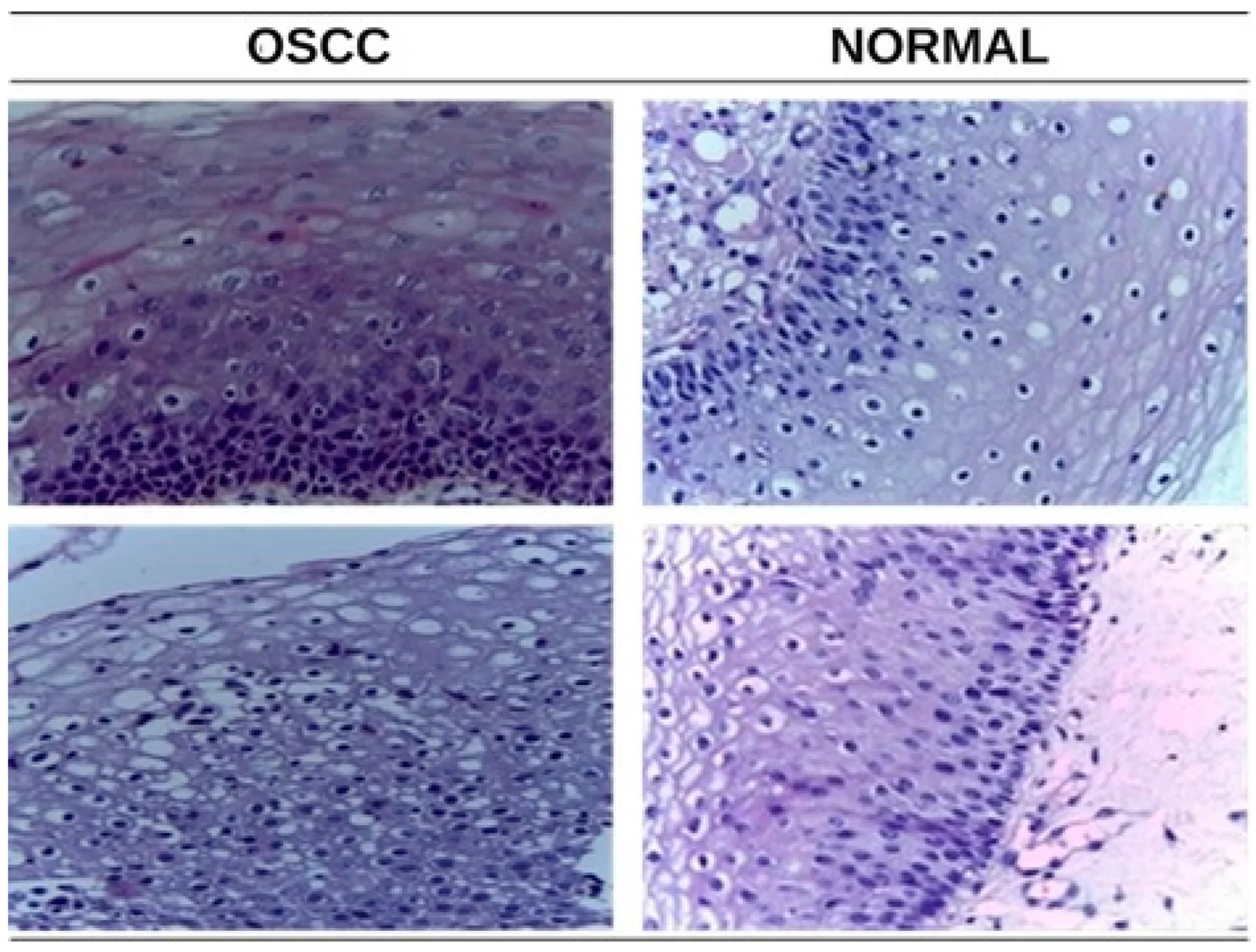
Sample images from the dataset.

A public dataset was used in this study, which was conducted to detect oral cancer using histopathological images. The dataset used contains 201 images of normal oral cavity epithelium at 400× magnification and 495 OSCC histopathological images. The images were obtained from Hematoxylin and Eosin (H&E) stained tissue slides collected, prepared, and cataloged by medical specialists from 230 patients using a Leica ICC50 HD microscope (Leica Microsystems, Wetzlar, Germany) [18]. Sample images from the dataset are presented in Figure 1.

To obtain comparable results and measure the performance of the models more objectively, the original dataset was used in the study. No data augmentation was performed in the article.

### 2.2. Baseline CNN Models

#### 2.2.1. ViTB16 Model

The Vision Transformer (ViT) is an architecture that processes images by dividing them into a series of segments, unlike traditional convolutional neural networks (CNNs), and is based entirely on the Self-Attention mechanism. Proposed by Dosovitskiy et al., the Base version of this model has a 12-layer Transformer encoder structure [19]. It divides the input image into 16 × 16 pixel sections. The model was trained on the more comprehensive ImageNet-21k dataset, consisting of 21,000 classes, instead of the standard ImageNet-1k. This training enables, the model to capture richer features in specialized areas such as histopathological images and demonstrate higher generalization success.

#### 2.2.2. ViTL16 Model

The ViTL16 architecture has a much deeper and wider neural network capacity compared to the standard Base model. It is designed to capture details in complex datasets. It contains 24 Transformer blocks and 16 attention heads. This structure significantly increases the model’s learning capacity with the increase in the number of parameters. By processing 16 × 16 sections and synthesizing local and global context, the model has become a powerful feature extractor, benefiting from the great visual diversity in the ImageNet-21k dataset. Pre-training strategies and regulation techniques are used in this architecture to counter the risk of overfitting in large models. These techniques ensure high accuracy rates in medical image analysis.

#### 2.2.3. ClipViTBase32 Model

Developed by OpenAI, CLIP (Contrastive Language-Image Pre-training) is a multi-layered structure that learns by associating visual data with text descriptions. This model, presented by Radford et al., uses the ViT-Base backbone [20]. It processes the image in larger sections of 32 × 32 dimensions. The fundamental difference in the model is that instead of classic label-based training, it is trained on the semantic relationship between image and text pairs using a contrastive loss function. This approach enables the model to learn visual patterns and their semantic context. Thus, it offers strong classification performance with transfer learning capability on limited datasets.

#### 2.2.4. ConvNextV2 Model

ConvNextV2 was developed to modernize convolutional neural networks and integrate the design structure of Transformer architectures into CNN models following the success of Vision Transformers. Proposed by Woo et al., this model is a fully convolutional architecture trained using a Masked Autoencoder (MAE)-based training strategy [21]. The Tiny variation is a lightweight version of the model that contains fewer parameters and has low computational cost but high performance. Thanks to its Global Response Normalization (GRN) layers, it increases inter-channel competition, thereby improving feature quality. ConvNextV2 is a powerful alternative to Transformer models in medical imaging in terms of both speed and accuracy.

#### 2.2.5. DeitBase16 Model

Data-efficient Image Transformers (DeiT) were developed to reduce the large data requirements needed to train models and achieve high performance with less data. This architecture, presented by Touvron et al., uses a different token that typically provides CNN information transfer in addition to the standard ViT structure [22]. This token enables the model to learn the incoming hard or soft labels. Operating at a 16 × 16 resolution, DeiT-Base is used in fields where labeled data is limited or imbalanced, such as histopathology, thanks to this unique strategy. This model combines the feature capture power of CNNs with the general context success of Transformers.

#### 2.2.6. Dinov2 Model

Dinov2 Base was developed by Meta AI. It is a Vision Transformer model trained using self-supervised learning without requiring labeled data. This approach, presented by Oquab et al., successfully models the semantic parts and textural structures of objects by learning discriminative features at the image level [23]. The Base variant was trained on a specially constructed large dataset (LVD-142M). The most distinctive feature of Dinov2 is that the features it produces are strong and discriminative. Even when a simple classifier (KNN or SVM) is added, this model can produce results that outperform supervised methods.

#### 2.2.7. ViTB32 Model

The ViTB32 architecture is a variation developed to improve the computational efficiency of the original Vision Transformer structure. The image is divided into larger pieces of 32 × 32 size instead of 16 × 16. By reducing the number of tokens entering the Transformer network by a quarter, it significantly reduces the quadratic complexity. This structural change allows the model to focus on coarser, more prominent details and speeds up inference times. Since it is trained on ImageNet-21k, it does not lose the overall structure and provides an efficient balance between computational load and accuracy in high-resolution texture analysis, offering a suitable infrastructure for fast recognition systems.

### 2.3. Proposed Model

**Figure 2:**
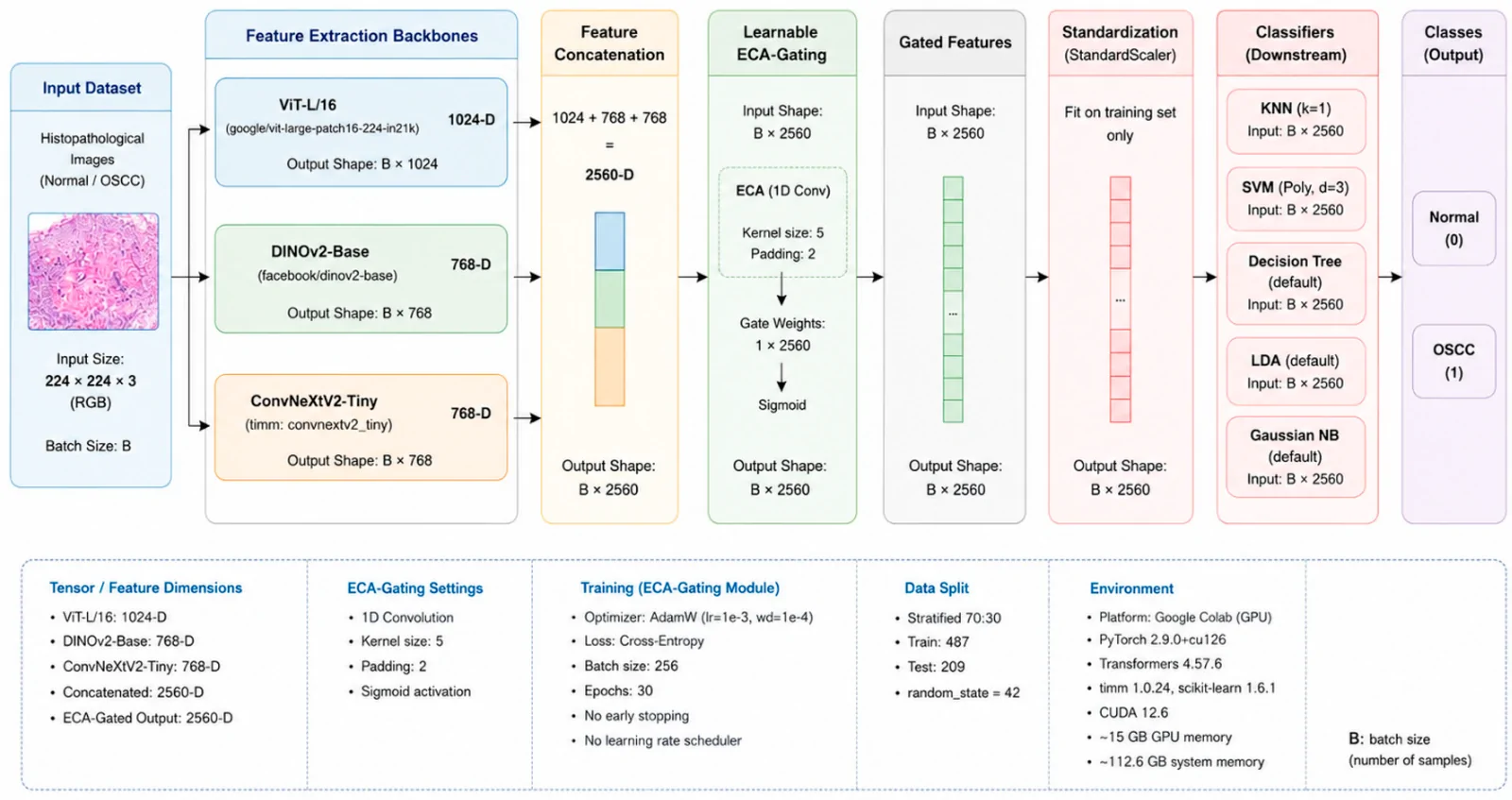
Proposed model.

This study on oral cancer detection proposes a hybrid classification approach based on combining features extracted from multiple pre-trained deep networks and then reweighting this combined representation in a controlled manner using a learnable gating mechanism. The proposed method consists of three main steps. The first step of the proposed model involves combining features obtained from the models that achieved the highest performance in the study. In the second step of the model, the discriminative components are highlighted using learnable gating based on Efficient Channel Attention (ECA). Finally, the gated representations are scaled and classified using various classical machine learning classifiers. The flowchart of the proposed model is presented in Figure 2.

Upon examining the proposed model, it is seen that in the first stage, the three models that yielded the most successful results among the models obtained from the seven different models used in the study were used as the basis. The features obtained from these models were combined. At this stage, the aim was to benefit from the complementary representation power of different architectures and to obtain a richer feature space without being dependent on a single backbone.

The contribution of each component of the combined features to classification may not be equal. Therefore, the combined features are passed through a learnable gating block based on Efficient Channel Attention (ECA), which is a lightweight and stable mechanism. The proposed ECA-Gate uses 1D convolution instead of the multi-layer perceptron (MLP) structure in classic SE to produce attention weights at the channel (feature dimension) level. This reduces the number of parameters and computational cost.

First, a “squeeze” representation is obtained by averaging along the batch axis, and this representation is converted into a gate vector in the range [0, 1] for each feature dimension using 1D convolution + sigmoid activation. Finally, this gate vector is multiplied by the feature matrix element-wise (channel-wise gating) to emphasize discriminative features and suppress less useful components.

To enable this gating mechanism to learn in a class-sensitive manner, an auxiliary linear classification head was used during training in addition to the gate block. This structure was implemented in the GateTrainer class. During training, the gate and head are optimized together; once training is complete, the head is discarded, and only the “gated” features produced by the gate block are passed to the next stage. This design enables the gating function to be learned directly through supervised loss, creating a kind of “learnable feature selection/reweighting” effect.

Since deep features can have different distributions, Gated Features are standardized before classical classifiers. For this purpose, StandardScaler is applied to the training set, and the same transformation is applied to the test set. This step contributes to the more stable operation of distance-based or margin-based classifiers in particular. The final step of the proposed model is the classification step. In this step, five different classifiers accepted in the literature are used.

## 3. Experimental Results

The experiments were conducted using a stratified 70:30 train–test split, resulting in 487 training and 209 test images, with a fixed random seed of 42. The pre-trained backbone networks were used as fixed feature extractors without fine-tuning. In the proposed framework, the 1024-, 768-, and 768-dimensional features extracted from ViT-L/16, DINOv2-Base, and ConvNeXtV2-Tiny, respectively, were concatenated to obtain a 2560-dimensional feature representation. The ECA-Gated module was trained for 30 epochs using AdamW with a learning rate of 1 × 10^−3^, weight decay of 1 × 10^−4^, cross-entropy loss, and a batch size of 256. All experiments were conducted in the Google Colaboratory environment with GPU acceleration, and a fixed random seed of 42 was used to support reproducibility. In this study, conducted to enable the detection of oral cancer from histopathological images, an ECA-based hybrid model is proposed. The proposed model is also used in feature fusion. To compare the performance of the proposed model, feature extraction was performed using seven different CNN and ViT architectures, and the features obtained were classified using five different classifiers. It was observed that the proposed model produced more successful results than the models and classifiers used in the study. Different evaluation metrics were used to compare the performance of the proposed model and the other models used in the study. These metrics are Accuracy (ACC), Error Rate, Weighted Precision, Weighted Recall, and Weighted F1 Score. Furthermore, in this section, the confusion matrices and performance evaluation metrics obtained by classifying the feature maps obtained from different models in different classifiers are classified separately.

### 3.1. Results of the ViTB16 Model

**Figure 3:**
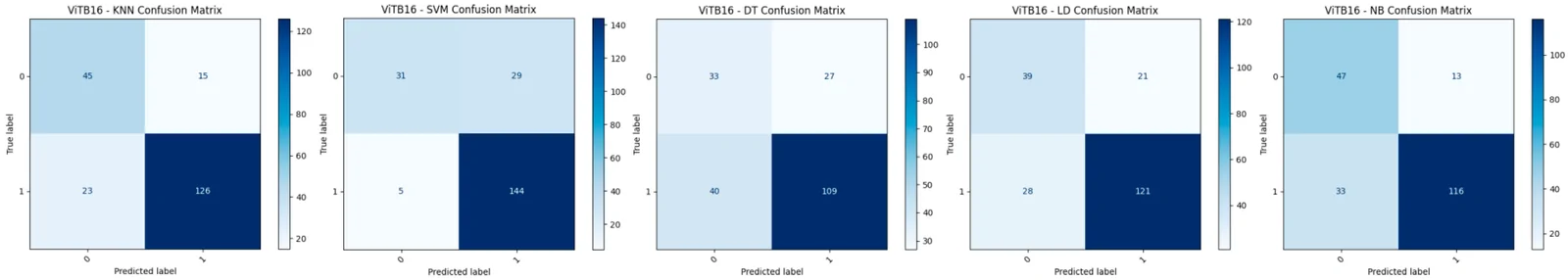
Confusion matrices obtained by classifiers for features obtained in the ViTB16 model.

The first model used in our study for the automatic detection of oral cancer is ViTB16. The feature maps obtained with the ViTB16 model were classified using five different classifiers accepted in the literature. The confusion matrices obtained are presented in Figure 3.

When examining Figure 3, it is seen that the SVM and KNN classifiers are more successful compared to other classifiers. In the SVM matrix, it is seen that 144 test images belonging to the OSCC class were correctly predicted and 5 test images were detected as false negatives. It is seen that the DT and NB classifiers confuse normal tissue samples with cancerous tissue. The false detection rates of these classifiers are higher. The performance metrics of the feature maps obtained with the ViTB16 model in the classifiers are presented in Table 1.

When evaluating the performance metrics provided in Table 1, it is observed that the SVM classifier demonstrates the highest success with an accuracy rate of 83.73% and an F1 score of 82.30%. The KNN classifier also shows consistent performance, following SVM with an accuracy rate of 81.82%. The DT classifier, however, remained at a low accuracy rate of 67.94%. It is seen that the feature maps extracted by the ViTB16 model are difficult to distinguish with simple decision boundaries. This indicates that complex hyperplanes are needed to overcome this situation.

### 3.2. Results of the ViTL16 Model

**Figure 4:**
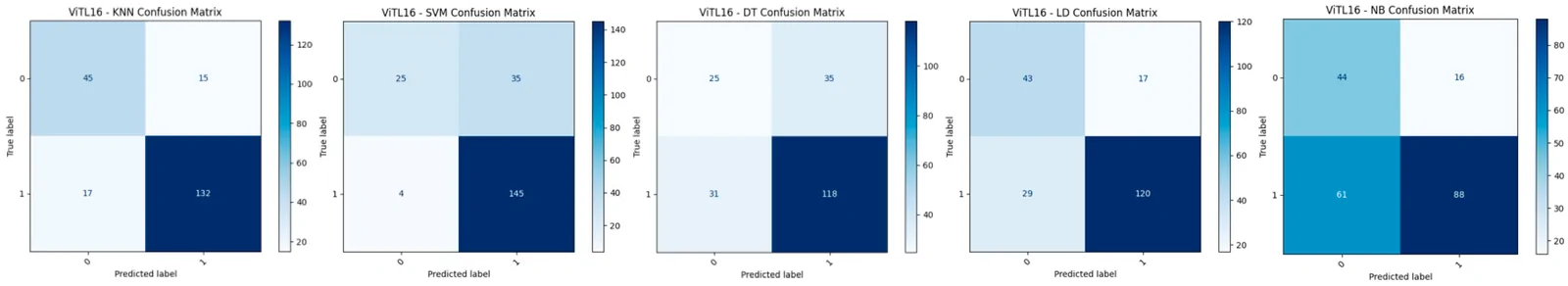
Confusion matrices for the ViTL16 model.

The second model used in our study for the automatic detection of oral cancer is ViTL16. The feature maps obtained with the ViTL16 model were classified using five different classifiers accepted in the literature. The confusion matrices obtained are presented in Figure 4.

When examining the confusion matrices presented in Figure 4 for this model, which has a larger architecture, it is seen that the KNN classifier produced the most successful results in class separation. In the confusion matrix obtained with the KNN classifier, 45 normal and 132 OSCC test images were correctly classified. It is understood that the number of misclassifications is low compared to other methods. In the confusion matrices of the NB and LD classifiers, a distinct weakness is noticeable, particularly in the detection of normal tissues. This indicates that the model can produce features that may create noise for some simple probabilistic classifiers. The performance metrics of the classifiers with the feature maps obtained with the ViTL16 model are presented in Table 2.

When examining the performance values in Table 2, it is seen that the KNN classifier is the most successful classifier with an accuracy rate of 84.69% and a Weighted F1 Score of 84.76%. SVM ranks second with 81.34%, while the NB classifier achieves a lower performance with an accuracy rate of 63.16%. These results show that the feature extraction provided by the structure of the ViTL16 model is processed more efficiently with KNN approaches.

### 3.3. Results of ClipViTBase32

**Figure 5:**
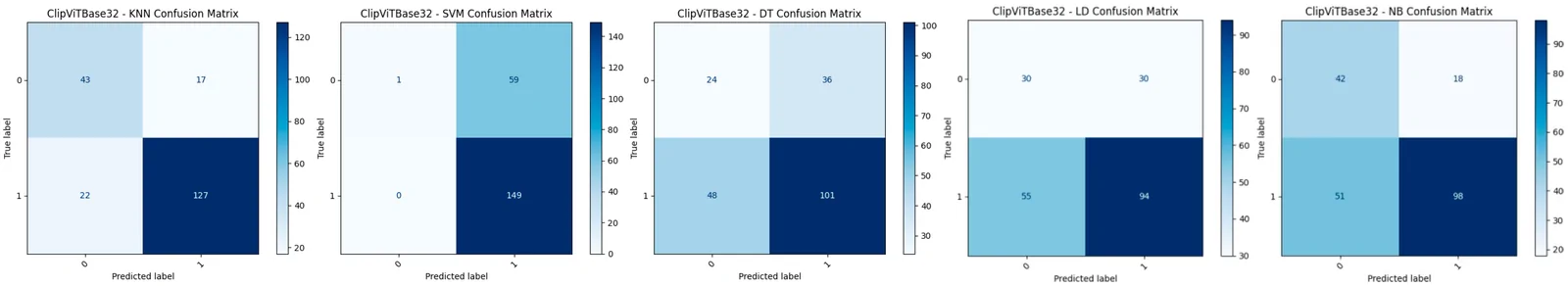
Confusion matrices obtained with different classifiers for the CLIP ViT Base Patch32 model.

Another model used in our study for the automatic detection of oral cancer is ClipViTBase32. The feature maps obtained with the ClipViTBase32 model were classified using different classifiers accepted in the literature. The confusion matrices obtained with these classifiers are presented in Figure 5.

When examining the confusion matrices presented in Figure 5 for the ClipViTBase32 architecture, it is understood that its effect on histopathological images varies depending on the classifier. The confusion matrix of the KNN classifier shows a partially balanced distribution and correctly identified 43 normal and 127 cancerous tissues. A serious imbalance and a class collapse-like situation are observed in the SVM matrix. In particular, the high error rates in the SVM and LD matrices indicate that the linear separability of the features extracted by the model is weak. The performance metrics of the feature maps obtained with the ClipViTBase32 model in the classifiers are presented in Table 3.

The obtained performance data shows that the overall success of the ClipViTBase32 model is lower than other ViT variations. The highest performance shown in Table 3 is provided by the KNN classifier with an accuracy rate of 81.34%. The failure of algorithms such as SVM (71.77%), DT (59.81%), and LD (59.33%) increased the model’s error rate. The weighted F1 score dropping to the 60% range indicates that this model may be insufficient for specific medical imaging tasks.

### 3.4. Results of ConvNextV2

**Figure 6:**
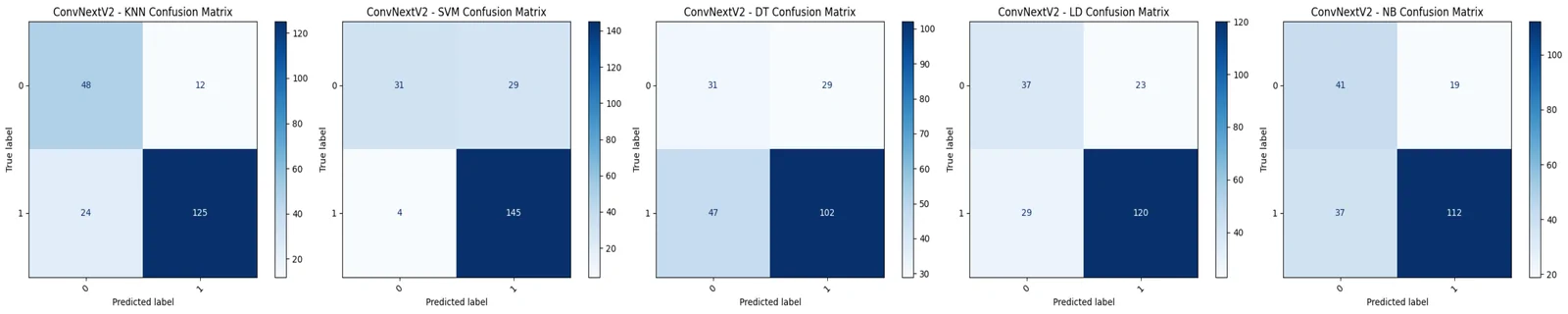
Confusion matrices for the ConvNextV2 model.

Another model used in our study for the automatic detection of oral cancer is ConvNextV2. Feature maps obtained with the ConvNextV2 model were classified using different classifiers accepted in the literature. The confusion matrices obtained in these classifiers are presented in Figure 6.

The confusion matrices of the convolutional neural network-based ConvNextV2 model are shown in Figure 6. The confusion matrices clearly reflect the model’s success in capturing local tissue features. The number of correct predictions is high in both the confusion matrices of the SVM and KNN classifiers. In the SVM matrix, 145 OSCC test images were correctly detected, while only 4 test images were missed. This indicates that the low false negative rate in the matrices demonstrates the model’s reliable sensitivity in cancer detection. The CNN architecture successfully transfers morphological changes into the feature space. The performance metrics of the feature maps obtained with the ConvNextV2 model in the classifiers are presented in Table 4.

When evaluating the performance results presented in Table 4, the SVM classifier achieved the highest accuracy of 84.21%, with a weighted precision of 84.84% and a weighted F1 score of 82.74%. The LD classifier, which showed the lowest performance, achieved an accuracy value of 75.12%. This indicates that the features produced by the ConvNext V2 Tiny model are generally of high quality and are discriminative regardless of the classifier.

### 3.5. Results of DeitBase16

**Figure 7:**
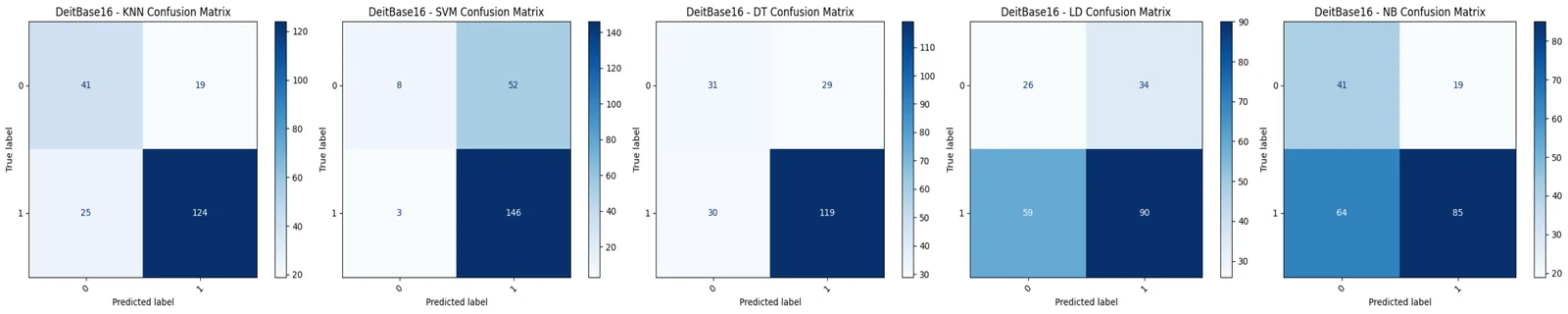
Confusion matrices for the classification results of the DeitBase16 model.

Another model used in our study for the automatic detection of oral cancer is DeitBase16. The feature maps obtained with the DeitBase16 model were classified using different classifiers accepted in the literature. The confusion matrices obtained in these classifiers are presented in Figure 7.

When examining the confusion matrices of the DeiTBase16 model, it reveals significant inconsistencies among the classifiers. As shown in Figure 7, the confusion matrix of the KNN classifier has produced an acceptable result by correctly separating 41 normal and 124 cancerous test images. However, the confusion matrix of the SVM classifier shows a significant drop in the detection of the normal class, and the model tends to predict almost all samples as cancerous. This imbalance indicates that the information provided by the distillation token cannot be effectively used by some classifiers and that the decision boundaries have become blurred. The performance metrics obtained for the relevant model are presented in Table 5.

According to the performance metrics in Table 5, the model achieved its best result with a 78.95% accuracy rate using the KNN classifier. SVM remained at 73.68%, while the LD classifier dropped to an accuracy rate close to chance, at 55.50%. This indicates that the model’s generalization ability is limited. The weighted F1 score also remained at 79.23% at best, confirming that the DeiT architecture is less efficient than other Transformer models for this specific dataset.

### 3.6. Results of Dinov2 Model

**Figure 8:**
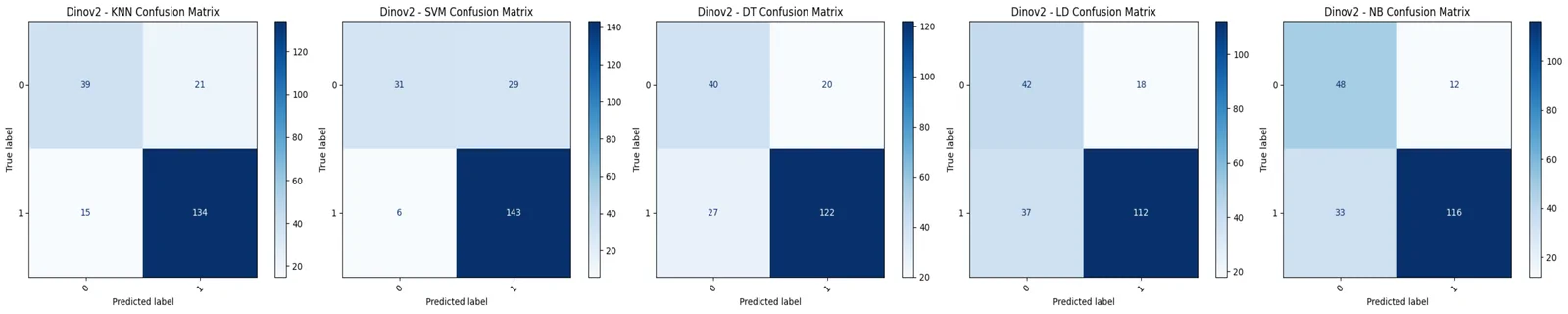
Confusion matrices for the Dinov2 model.

Another model used in our study for the automatic detection of oral cancer is Dinov2. The feature maps obtained with the Dinov2 model were classified using different classifiers accepted in the literature. The confusion matrices obtained in these classifiers are presented in Figure 8.

The confusion matrices in Figure 7 clearly reflect the model’s success in capturing local tissue features. The number of correct predictions is high in both the SVM and KNN classifiers’ confusion matrices. In the SVM matrix, 143 OSCC test images were correctly detected, while only 6 test images were missed. This situation indicates that the low false negative rate in the matrices and the model’s sensitivity in cancer detection are at a reliable level. The performance metrics obtained in the relevant model are presented in Table 6.

According to the performance metrics in Table 6, the model achieved its best result with an accuracy rate of 83.25% in the SVM classifier. KNN remained at 82.78%, while the LD classifier dropped to an accuracy rate of 73.68%. This indicates that the model’s generalization ability is limited. The weighted F1 score also remained at 82.49% at best, indicating that the features produced by the Dinov2 architecture model are generally of high quality and have discriminative properties independent of the classifier.

### 3.7. Results of the VITB32 Model

**Figure 9:**
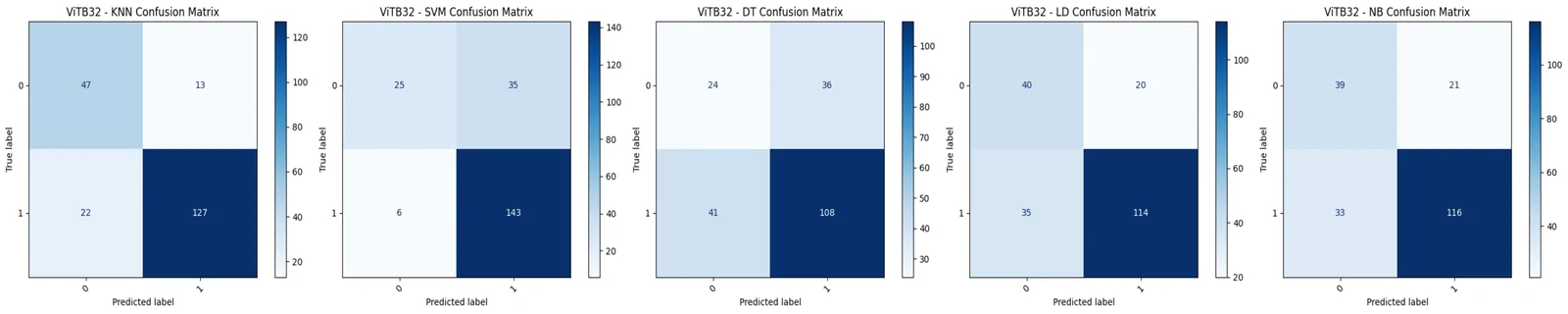
Confusion matrices for the ViTB32 model.

Another model used in our study for the automatic detection of oral cancer is VITB32. The feature maps obtained with the VITB32 model were classified using different classifiers accepted in the literature. The confusion matrices obtained in these classifiers are presented in Figure 9.

The confusion matrices in Figure 9 clearly reflect the model’s success in capturing local tissue features. The number of correct predictions is high in both the SVM and KNN classifiers’ confusion matrices. In the SVM matrix, 143 OSCC test images were correctly detected, while only 6 test images were missed. This indicates that the low false negative rate in the matrices demonstrates the model’s reliable sensitivity in cancer detection. The performance metrics obtained for the relevant model are presented in Table 7.

According to the performance metrics in Table 7, the model achieved its best result with an accuracy rate of 83.25% in the KNN classifier. SVM remained at 80.38%, while the LD classifier dropped to an accuracy rate of 73.68%. This indicates that the model’s generalization ability is limited. The fact that the weighted F1 score also remained at 83.58% at best indicates that the features produced by the VitB32 architecture model are generally of high quality and are discriminative in nature, independent of the classifier.

### 3.8. Proposed Method

**Figure 10:**
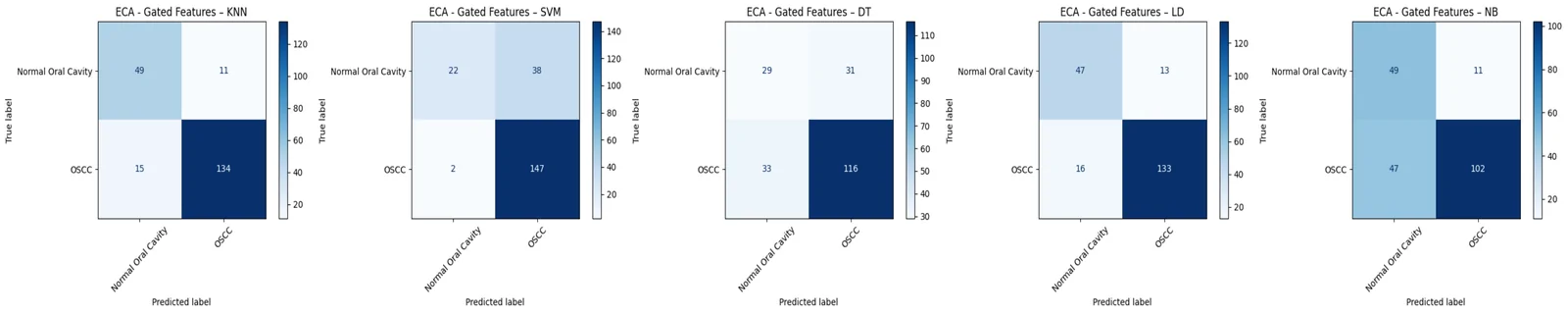
Confusion matrices for the proposed ViTL16 + Dinov2 (ECA—Gated Features) hybrid model.

A hybrid model is proposed in this study conducted for the detection of oral cancer. The proposed model is detailed in Section 2. In the first step of the proposed model, ViTL16 + Dinov2 models are used for feature extraction. The confusion matrices obtained in this step are presented in Figure 10.

An examination of the confusion matrices in Figure 10 reveals the success of the feature fusion strategy. The number of misclassifications in the matrices has decreased significantly compared to the pre-trained models. In particular, the KNN classifier correctly predicted 49 normal tissues and 134 OSCC cases, and the decrease in false negatives indicates an increase in the model’s sensitivity. The high success rate observed in the LD classifier matrix, unlike previous models, proves that the combined features can be linearly better discriminated. The performance evaluation metrics obtained in the first stage of the model are shown in Table 8.

When examining Table 8, the results obtained in the first step of the proposed hybrid model are better than those of the pre-trained models. As seen in Table 8, the KNN algorithm achieved high success with 87.56% accuracy and 87.68% F1 score. More importantly, the LD classifier, which failed in single models, has increased to an accuracy rate of 86.12% in this model. This increase shows that the proposed ECA (Gated Features) mechanism successfully synthesizes features from different models, thereby improving classification performance and model reliability.

**Figure 11:**
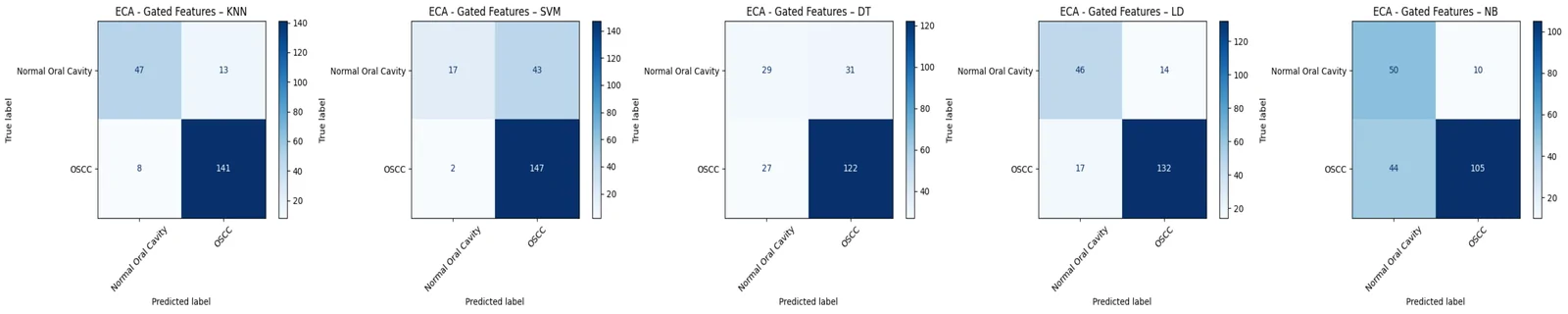
Confusion matrices for the proposed ViTL16 + Dinov2 + ConvNextV2 hybrid model.

In the second step of our proposed model, the ViTL16 + Dinov2 + ConvnextV2 models were used as the base. The confusion matrices obtained in the proposed model are presented in Figure 11.

The confusion matrices obtained in the proposed model demonstrate successful classification performance. In the KNN matrix, 47 normal and 141 OSCC samples were correctly classified. In particular, the LD matrix also provided high performance similar to KNN. This demonstrates that the complexity and representational power of the features provided by the model are at the highest level. The reduction in misclassifications supports the reliability factor, which is vital for clinical decision support systems. The performance evaluation metrics obtained in the proposed model are shown in Table 9.

When examining the performance metrics in Table 9, it is observed that this model achieves the highest performance compared to previous models. With the KNN classifier, an accuracy rate of 89.95%, an F1 score of 89.82%, and an error rate of only 10.05% were obtained. The balanced and high Precision and Recall values prove that the model does not show bias towards any class. These results suggest that, within the present experimental setting, combining the global representation capabilities of Transformer-based models with the local feature extraction capabilities of CNN-based models can improve the classification of normal oral epithelium and OSCC.

In the proposed model, the most successful results were obtained with the KNN classifier. Additional performance measurement metrics for this classifier are presented in Table 10.

Table 10 metrics provide a more comprehensive evaluation of the proposed model. The high sensitivity (94.63%) and PPV (91.56%) indicate strong OSCC detection performance, while the balanced accuracy (86.48%), MCC (0.7496), and Cohen’s kappa (0.7483) demonstrate consistent classification performance across the two classes.

## 4. Discussion

This study comprehensively evaluated various deep learning architectures and proposed hybrid methods for the automatic detection of oral cancer from histopathological images. The findings highlight the importance of architectural selection and feature fusion strategies in medical image analysis.

First, when the basic models were examined, it was found that ViT architectures generally yielded successful results. When the results were examined, it was seen that they remained in a similar performance range with modern CNN architectures such as ConvNext. This suggests that only Transformer or only CNN may not be sufficient in histopathological tissue analysis. This suggests that both structures are critically important. The low performance of DeiT and CLIP models, ranging from 60% to 78%, indicates that the training strategies or data efficiency of these models may be insufficient for specific histopathology datasets without fine-tuning.

The Dinov2 model demonstrated approximately 83% success without requiring labeled data, thanks to its unsupervised learning capability. This proves the model’s high feature extraction power. This finding supports why Dinov2 was chosen while forming the basis of the proposed hybrid model.

The most striking finding of the study is the performance of the proposed ViTL16 + Dinov2 + ConvNextV2 hybrid model. The accuracy rates of the individual models, which were stuck around 84%, have increased to 89.95% thanks to this hybrid structure. When the confusion matrices are examined, it is seen that this increase is not specific to only one class. Errors decreased for both normal tissue and OSCC detection. ConvNext’s ability to capture local morphological details, combined with ViT and Dinov2’s analysis of the tissue’s overall structure and semantic integrity, along with the ECA mechanism, reduced the model’s error rate to 10.05%. Even simple classifiers like LD, which failed in single models, achieved over 86% success with the features of the proposed model. This confirms that the generated feature space is linearly separable and successful.

Several limitations must be considered when interpreting the findings of this study. First, although the publicly available dataset contains 696 histopathological images from 230 patients, it lacks patient- and sample-level matching information to identify the patient or tissue sample to which the images belong. Due to these limitations, the dataset was partitioned at the image level. The possibility that images belonging to the same patient or tissue sample may be distributed between the training and test sets cannot be completely ruled out. Second, the model’s performance was evaluated using only a single publicly available dataset and a single training–test partitioning. Repeated patient-level validation or independent external validation was not performed in the study. Finally, the dataset was not compared with expert pathologists or clinically used computer-aided diagnostic systems. Future studies should focus on patient-level and multicenter independent external validation studies, repeated evaluations, controlled ablation, and integrity analyses to address these limitations. Explainable artificial intelligence methods can be used in conjunction with expert pathologist evaluations. This situation will allow for a more comprehensive evaluation of the clinical validity and applicability of the proposed approach, by assessing computational efficiency and directly comparing model performance with the diagnostic performance of expert pathologists.

## 5. Conclusions

Histopathological examination plays a central role in the diagnosis of oral cancer. Evaluating histopathological images in these examinations can be time-consuming and require specialized pathological expertise. In this study, a hybrid deep learning model was developed for the automated classification of normal oral epithelium and OSCC in histopathological images. The model integrates complementary feature representations derived from ViT-L/16, DINOv2, and ConvNeXtV2 via an ECA–Gated Features merging mechanism. Among the evaluated models, the proposed hybrid model achieved the highest accuracy of 89.95% in the current experimental environment. The findings demonstrate that combining feature representations obtained from convolutional, Transformer-based, and self-supervised learning models can provide complementary feature information in distinguishing normal oral epithelial tissue from OSCC. The results should be interpreted within the context of the current dataset and experimental design. In this study, patient-level discrimination and external validation were not possible, and robustness, explainability, computational efficiency, and direct comparisons with expert pathologists were not systematically evaluated. Further validation studies at the patient level, on independent and preferably multicenter datasets, are needed to determine the success of this method in different patient groups and its potential contribution to clinical decision support processes. Future studies should also investigate controlled robustness and explainability analyses, computational efficiency, and the model’s application to histopathological diagnoses such as tumor segmentation.

## Figures and Tables

**Table 1:** Performance metrics of the ViTB16 model on the test dataset.

Classifiers	ACC % (Test)	Error Rate % (Test)	Weighted Precision %	Weighted Recall %	Weighted F1 Score %
KNN	81.82	18.18	82.71	81.82	82.14
SVM	83.73	16.27	84.06	83.73	82.30
DT	67.94	32.06	70.12	67.94	68.78
LD	76.56	23.44	77.46	76.56	76.92
NB	77.99	22.01	80.97	77.99	78.77

**Table 2:** Performance metrics of the ViTL16 model on the test dataset.

Classifiers	ACC % (Test)	Error Rate % (Test)	Weighted Precision %	Weighted Recall %	Weighted F1 Score %
KNN	84.69	15.31	84.85	84.69	84.76
SVM	81.34	18.66	82.18	81.34	78.97
DT	68.42	31.58	67.80	68.42	68.09
LD	77.99	22.01	79.59	77.99	78.53
NB	63.16	36.84	72.35	63.16	64.91

**Table 3:** Performance metrics of the ClipViTBase32 model in classifiers.

Classifiers	ACC % (Test)	Error Rate % (Test)	Weighted Precision %	Weighted Recall %	Weighted F1 Score %
KNN	81.34	18.66	81.87	81.34	81.55
SVM	71.77	28.23	79.78	71.77	60.45
DT	59.81	40.19	62.13	59.81	60.79
LD	59.33	40.67	64.18	59.33	60.97
NB	66.99	33.01	73.19	66.99	68.49

**Table 4:** Performance metrics of the ConvNextV2 model in the classifier.

Classifiers	ACC % (Test)	Error Rate % (Test)	Weighted Precision %	Weighted Recall %	Weighted F1 Score %
KNN	82.78	17.22	84.19	82.78	83.20
SVM	84.21	15.79	84.84	84.21	82.74
DT	63.64	36.36	66.92	63.64	64.84
LD	75.12	24.88	75.92	75.12	75.46
NB	73.21	26.79	76.04	73.21	74.09

**Table 5:** Performance values of the DeiTBase16 model on the test set.

Classifiers	ACC % (Test)	Error Rate % (Test)	Weighted Precision %	Weighted Recall %	Weighted F1 Score %
KNN	78.95	21.05	79.65	78.95	79.23
SVM	73.68	26.32	73.45	73.68	66.46
DT	71.77	28.23	71.91	71.77	71.84
LD	55.50	44.50	60.53	55.50	57.30
NB	60.29	39.71	69.48	60.29	62.17

**Table 6:** Performance table showing the classification success of the Dinov2 model.

Classifiers	ACC % (Test)	Error Rate % (Test)	Weighted Precision %	Weighted Recall %	Weighted F1 Score %
KNN	82.78	17.22	82.37	82.78	82.49
SVM	83.25	16.75	83.32	83.25	81.87
DT	77.51	22.49	78.39	77.51	77.86
LD	73.68	26.32	76.68	73.68	74.59
NB	78.47	21.53	81.62	78.47	79.26

**Table 7:** Performance results of the ViTB32 model.

Classifiers	ACC % (Test)	Error Rate % (Test)	Weighted Precision %	Weighted Recall %	Weighted F1 Score %
KNN	83.25	16.75	84.23	83.25	83.58
SVM	80.38	19.62	80.43	80.38	78.13
DT	63.16	36.84	64.07	63.16	63.58
LD	73.68	26.32	75.96	73.68	74.45
NB	74.16	25.84	75.91	74.16	74.80

**Table 8:** Comparative performance results of the proposed ViTL16 + Dinov2 hybrid method.

Classifiers	ACC % (Test)	Error Rate % (Test)	Weighted Precision %	Weighted Recall %	Weighted F1 Score %
KNN	87.56	12.44	87.86	87.56	87.68
SVM	80.86	19.14	82.96	80.86	77.79
DT	69.38	30.62	69.69	69.38	69.53
LD	86.12	13.88	86.36	86.12	86.22
NB	72.25	27.75	79.01	72.25	73.54

**Table 9:** Performance metrics of the proposed hybrid model.

Classifiers	ACC % (Test)	Error Rate % (Test)	Weighted Precision %	Weighted Recall %	Weighted F1 Score %
KNN	89.95	10.05	89.81	89.95	89.82
SVM	78.47	21.53	80.84	78.47	74.18
DT	72.25	27.75	71.71	72.25	71.95
LD	85.17	14.83	85.42	85.17	85.27
NB	74.16	25.84	80.36	74.16	75.35

**Table 10:** Additional performance measurement metrics in the KNN classifier of the proposed model.

Accuracy	Sensitivity	Specificity	Positive Predictive Value (PPV)
89.95	94.63	78.33	91.56
Negative Predictive Value (NPV)	Balanced Accuracy	Matthews Correlation Coefficient (MCC)	Cohen’s Kappa
85.45	86.48	0.7496	0.7483

## Data Availability

The dataset supporting the conclusions of this article is available in the Mendeley repository at https://data.mendeley.com/datasets/ftmp4cvtmb/1 (accessed on 1 February 2026) [18].
